# Percutaneous intervention for salvage of non-maturing arteriovenous fistulas: Which is the better approach, arterial or venous?

**DOI:** 10.1371/journal.pone.0238788

**Published:** 2020-09-29

**Authors:** Sang Min Lee, Jae Boem Na, Ho Cheol Choi, Jung Ho Won, Ji Eun Kim, Ji Hoon Shin, Hyun Oh Park, Sung Eun Park

**Affiliations:** 1 Department of Radiology, Gyeongsang National University College of Medicine and Gyeongsang National University Hospital, Jinju, Republic of Korea; 2 Department of Radiology and Research Institute of Radiology, Asan Medical Center, University of Ulsan College of Medicine, Seoul, Republic of Korea; 3 Thoracic and Cardiovascular Surgery, Gyeongsang National University School of Medicine and Gyeonsang National University Hospital, Jinju, Republic of Korea; 4 Department of Radiology, Gyeongsang National University College of Medicine and Gyeonsang National University Changwon Hospital, Changwon, Republic of Korea; University Magna Graecia of Catanzaro, ITALY

## Abstract

**Objectives:**

To evaluate the efficacy and long-term patency of endovascular treatment for non-maturing native arteriovenous fistulas according to the approach route (arterial vs. venous).

**Methods:**

Eighty-five patients underwent percutaneous transluminal angioplasty for non-maturing fistulas (63 radiocephalic and 22 brachiocephalic) between 2010 and 2019. Outcome variables such as procedural success, complications, and primary and secondary patency rates were analyzed from the patients’ demographic, angiographic, clinical, and hemodialysis records according to the approach route (venous access group, n = 53 and arterial access group, n = 32). The Kaplan-Meier method was used to analyze the patency rates.

**Results:**

The mean duration from fistula creation to fistulography was 78.4±51.4 days (range, 1–180 days). The anatomical and clinical success rates were 98.8% and 83.5%, respectively. Lesions were most commonly located at the juxta-anastomosis (55.3%). Accessory cephalic veins were observed in 16 patients. The primary patency rates were 83.9%, 71.9%, and 66.3% and the secondary patency rates were 98.6%, 95.9%, and 94.2% at 3 months, 6 months, and 1 year, respectively. The degree of hypertension (P = 0.023), minimal preoperative vein size (P = 0.041), and increment in postoperative vein diameter were higher in the venous access group than in the arterial access group (P<0.01). The frequency of using cutting balloons (P = 0.026) and complication rate were higher in the arterial access group than in the venous access group (arterial access: 1 major, 8 minor; venous access: 4 minor; P = 0.015).

**Conclusions:**

Aggressive evaluation and endovascular therapy can salvage most non-maturing fistulas. Transradial and distal radial approaches can be effective even for challenging lesions.

## Introduction

A native arteriovenous fistula (AVF) provides superior vascular access for hemodialysis than that of synthetic grafts owing to the longer patency duration, fewer infections, and lower mortality [1]. However, approximately 30% of native AVFs do not mature to enable successful hemodialysis [2, 3]. Percutaneous transluminal angioplasty (PTA) has been used for the treatment of mature AVF malfunctions with high clinical outcomes, whereas non-maturing fistulas were previously considered contraindications for any endovascular approach because they are fragile, may be difficult to palpate, and are accompanied by complex accessory veins. The optimal approach for immature fistulas remains controversial. Various techniques have been adopted for PTA for immature fistulas, including proximal and/or distal vein [4–6], brachial artery [7], and radial artery punctures [8, 9]. Both transvenous and transarterial approaches have advantages and disadvantages. The results of endovascular management are variable. Therefore, comparison of the clinical outcomes and long-term patency based on the approach routes is most useful after performing PTA for immature fistulas at the same center, with identical procedure techniques, definitions, and analysis criteria for both groups (arterial access vs. venous access). Herein, we aimed to evaluate the efficacy and long-term patency of endovascular therapy for non-maturing fistulas and compared the results obtained from the arterial and venous access approaches.

## Materials and methods

### Patient selection

**Fig 1:**
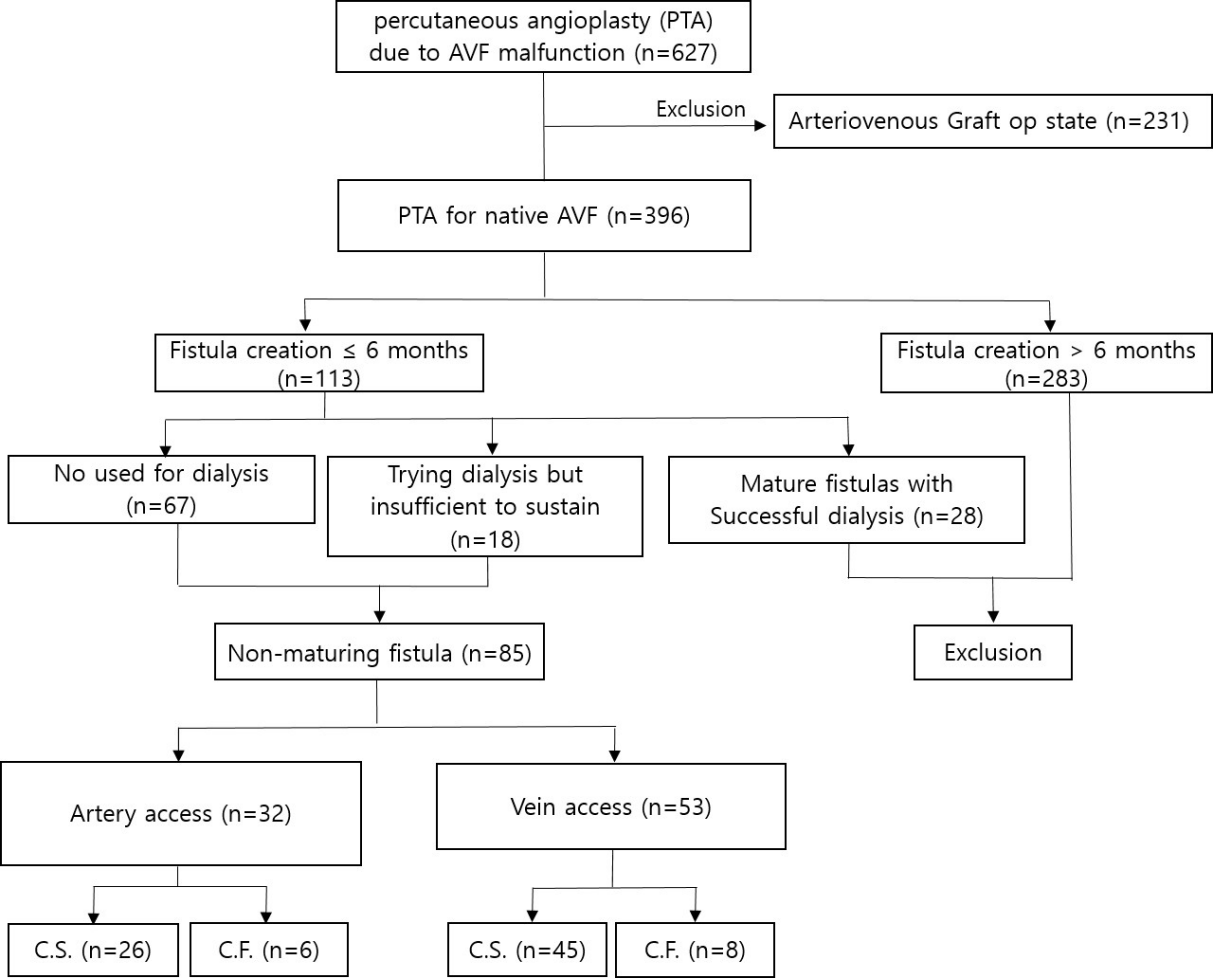
Algorithm of patient selection. C.S = clinical success, C.F = clinical failure.

The institutional review board of University of Gyeonsang National University Hospital approved this retrospective review of the patients’ medical and imaging records and waived the requirement for informed consent (IRB No. 2019-08-017). A total of 627 patients who underwent PTA due to AVF malfunction between January 2010 and June 2019 were registered in a dedicated database. We excluded 231 patients who underwent arteriovenous grafting and the following patients (from among the remaining 396 patients) with native AVF: those with fistulas created >6 months before (n = 293) and AVF malfunction after successful dialysis even if the fistula was created <6 months before (n = 28). In our practice, native fistulas are evaluated 4–6 weeks after creation by the surgical or nephrology team. The fistula is allowed to mature if it is clearly visible and shows no abnormal physical findings until it is ready for use (usually 3 months after creation). The patient was referred to an interventional radiologist for ultrasonography and diagnostic fistulography, and percutaneous salvage was attempted if the fistula did not mature sufficiently. Some patients were referred to the Department of Interventional Radiology after a much longer period (>3 months after fistula creation), as they delayed their hospital visit or showed ambiguous maturation, which needed immediate action. Therefore, the immature fistulas in this study included fistulas that had been created <6 months before and had never been used (n = 67) or those that had been used but were insufficient to sustain hemodialysis (n = 18). The patient recruitment process is summarized in Fig 1.

### Procedure technique

**Fig 2:**
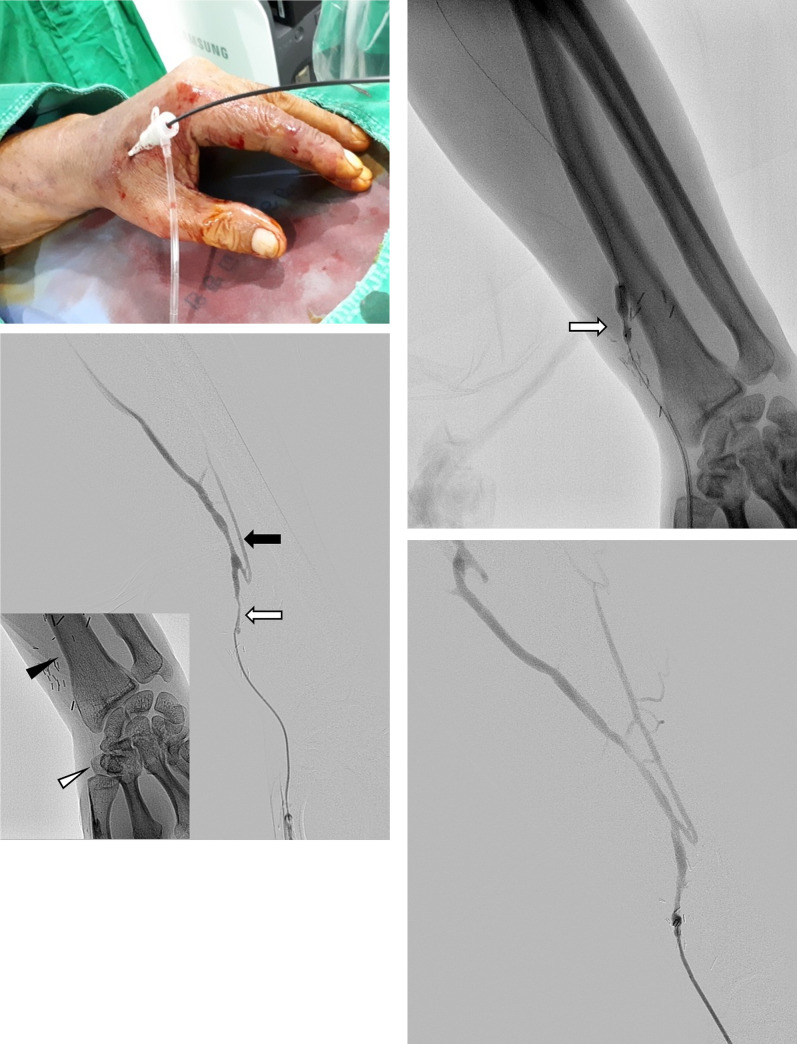
A 64-year old man with a non-maturing left radiocephalic fistula created 89 days before the procedure. Transvenous access was not feasible because the draining vein was small. Moreover, the transradial approach was not appropriate because of the short distance between the anastomosis site and the possible puncture site of the radial artery. (a) The 5-French introducer sheath inserted in the distal radial artery through the anatomical snuffbox. (b) The initial angiogram shows stenosis at the juxta-anastomosis vein (white arrow) with poor maturation of the cephalic vein. An accessory cephalic vein can also be observed (black arrow). The puncture site was over the trapezium (white arrowhead, inset), and sufficient length was obtained for the sheath up to the anastomosis site (black arrowhead, inset). (c) Severe stenosis did not improve even after PTA with a 5-mm-diameter balloon. Thus, a 5-mm-diameter cutting balloon was necessary to efface the waist (arrow). (d) The postoperative angiogram shows improvement in the stenosis. The patient underwent ligation of the accessory vein after PTA, and the fistula was used for 16 months without further intervention. PTA: percutaneous transluminal angioplasty.

The puncture site for diagnostic fistulography was determined after physical examination and ultrasonography for AVF. All the procedures were performed by one of three interventional radiologists with 5–16 years of clinical experience. We routinely attempted the retrograde venous approach first in 53 patients. However, an antegrade radial or brachial arterial approach was needed in 32 patients because of the difficulty of puncturing the small vein and/or multiple lesions needing two punctures for simultaneous use of the antegrade and retrograde approaches. Transradial arterial access was used if the radial artery was palpable, with a minimum diameter of 2 mm on ultrasonography, and the Allen test was negative. The skin area, usually 2-cm peripheral to the site of the anastomosis of the radial artery and cephalic vein, was sterilized and anesthetized [10]. If the anastomosis was located <2 cm proximal to the styloid process of the radius, distal radial artery (DRA) access was considered after performing the Barbeau test (Fig 2) [11]. The radial artery was accessed under ultrasonographic guidance using a 5—or 6-French transradial kit (Prelude, Merit Medical, Utah, USA; or Radiofocus, Terumo, Tokyo, Japan). After ensuring access, heparin 3,000 IU and nitroglycerin 200 μg were administered through the sheath. Diagnostic angiography was performed for examination, and the steno-occlusive lesions in the arterial or venous segment were bypassed using a 0.035-inch hydrophilic guidewire (GlideWire, Terumo, Tokyo, Japan) and 5-French Kumpe catheter (Cook Medical, Bloomington, IN, USA) under roadmap fluoroscopy. The lesions were treated with serial dilatations using a 3- to 10-mm Mustang balloon dilatation catheter (Boston Scientific, Galway, Ireland). Stenoses of the anastomotic or juxta-anastomotic area, peripheral vein, and central vein were treated with 3- to 6-mm-, 4- to 7-mm-, and 10-mm-diameter balloons, respectively. The balloon was inflated gradually until the disappearance of the waist caused by stenosis, usually for 1 min at a time. A cutting balloon (Boston Scientific, Galway, Ireland) was used if the balloon could not dilate the lesion. If necessary, the accessory cephalic veins were embolized with coil/microcoils or marked on the skin in preparation for surgical ligation. Hemostasis of the access site was achieved with a pneumatic radical compression device (PreludeSYNC; Merit Medical, Utah, USA). The fistula was allowed to mature after the procedure until it was ready for use. We checked the patency and maturation grade of the fistula with physical examination and/or Doppler ultrasonography at intervals of 1 to 3 months.

### Evaluation of data and definitions

Anatomical success was defined as the achievement of stenosis with a residual diameter of <30% and flow restoration, in accordance with the reporting standards set by the Society of Interventional Radiology (SIR) [12]; clinical success, as successful dialysis (blood flow rate of >350 mL/min) using the treated fistula for at least one session after the intervention [6]; procedure time, as the time interval from puncture to the final angiography; primary patency, as the interval between the primary and repeat interventions; and secondary patency, as the interval after intervention until the access was surgically declotted, revised, or abandoned, or the time when the patient received a renal transplant. Lesion locations were classified according to the system used by Clark et al. [13]. The juxta-anastomosis segment is in the initial 5 cm of the AVF starting at the arterial anastomosis. The peripheral vein was defined as the venous outflow tract of the AVF that starts proximal to the juxta-anastomosis segment and ends at the distal edge of the subclavian vein. The subclavian vein, innominate vein, or superior vena cava was referred to as the central vein. The lesion length was measured on angiography. The accessory vein was defined as a branch of the main venous channel that comprised the fistula. The minimal preoperative and postoperative sizes of the veins were usually measured using ultrasonography. Fistulography was used if ultrasonography was not available. Complications were categorized as major or minor according to the SIR Standards of Practice Committee guidelines [14].

### Statistical analysis

The Kaplan-Meier method was used to analyze the patency, and statistical difference was determined using the log-rank test. Outcomes were compared according to the approach route (arterial vs venous). Continuous variables with normal and non-normal distributions were analyzed with the Student *t* and Mann-Whitney *U* tests, respectively. Categorical data were assessed using the Fisher exact or χ^2^ test. All statistical analyses were performed using the Statistical Package for the Social Sciences version 21.0 for Windows (SPSS, Inc., Chicago, IL, USA).

## Results

### Patient characteristics

The baseline characteristics of the 85 patients (44 men and 41 women; mean age: 62.8±12.6 years) who underwent PTA due to immature AVFs are summarized in Table 1. Of the 85 patients, 68 (80%) had diabetes and 69 (81%) had hypertension. Twenty-one (25%), 10 (12%), 9 (11%), and 6 patients (7%) had past histories of coronary artery disease, cerebrovascular disease, and peripheral vascular disease, and present history of dyslipidemia, respectively. The mean blood urea nitrogen, creatinine, and potassium levels were 56.1 mg/dL, 5.5 mg/dL, and 4.4 mmol/L, respectively. The mean body mass index during fistula creation was 22.8±3.8 kg/m^2^. The minimal preoperative and postoperative vein sizes were 2.78±0.61 and 5.66±1.62 mm, respectively. The postoperative increment in vein diameter was 2.96±1.54 mm. Sixty-three patients (74%) had radiocephalic AVFs; and 22 (26%), brachiocephalic AVFs. Of the 22 patients with radiocephalic AVF who were treated with arterial access, 18 underwent radial artery puncture and 4 underwent DRA puncture through the anatomical snuffbox. Of the 10 patients with brachiocephalic AVF and arterial access, 8 underwent radial artery puncture and 2 underwent brachial artery puncture. Fistulas were located on the right arm in 18 patients and on the left arm in 67 patients. Five patients (6%) underwent repeated fistula creation surgeries. The mean time from fistula creation to endovascular salvage (fistula age) was 78.4±51.4 days (range, 1–180 days).

**Table 1:** Patient baseline characteristics.

Characteristic	Vein access (n = 53)	Artery access (n = 32)	Total (n = 85)	P value
Age (years)	61.4±12.2	64.9±13.1	62.8±12.6	0.173
Gender, male	26	18	44	0.635
Current/former smokers	17	16	33	0.100
Diabetes	44	24	68	0.371
Hypertension	47	22	69	0.023*
Coronary artery disease	21	8	29	0.168
Cerebrovascular disease	10	8	18	0.503
Peripheral vascular disease	9	2	11	0.153
Dyslipidemia	6	1	7	0.183
Lab finding (BUN/Cr/K)	52.1/5.5/4.4	62.8/5.6/4.4	56.1/5.5/4.4	>0.05
BMI	23.3±3.9	21.9±3.5	22.8±3.8	0.088
Preoperative minimal vein size (mm)	2.79±0.62	2.56±0.58	2.78±0.61	0.041*
Postoperative increment in diameter of vein	3.53±1.49	2.02±1.13	2.96±1.54	<0.01*
AVF type (puncture vessels)				0.380
*Radiocephalic*	41(all cephalic)	22 (18 RA / 4 distal RA)	63	
*Brachiocephalic*	12 (all cephalic)	10 (8 RA/2 BA)	22	
Right-sided fistula	12	6	18	0.670
Repeated fistula creation operation	1	4	5	0.064
Fistula age (day) (range)	80.9±50.5	74.3±53.5	78.4±51.4	0.474

BUN, blood urea nitrogen; Cr creatinine; K potassium; BMI, body mass index (kg/m^2^); BA, brachial artery; RA, radial artery.

### Outcome of PTA

**Fig 3:**
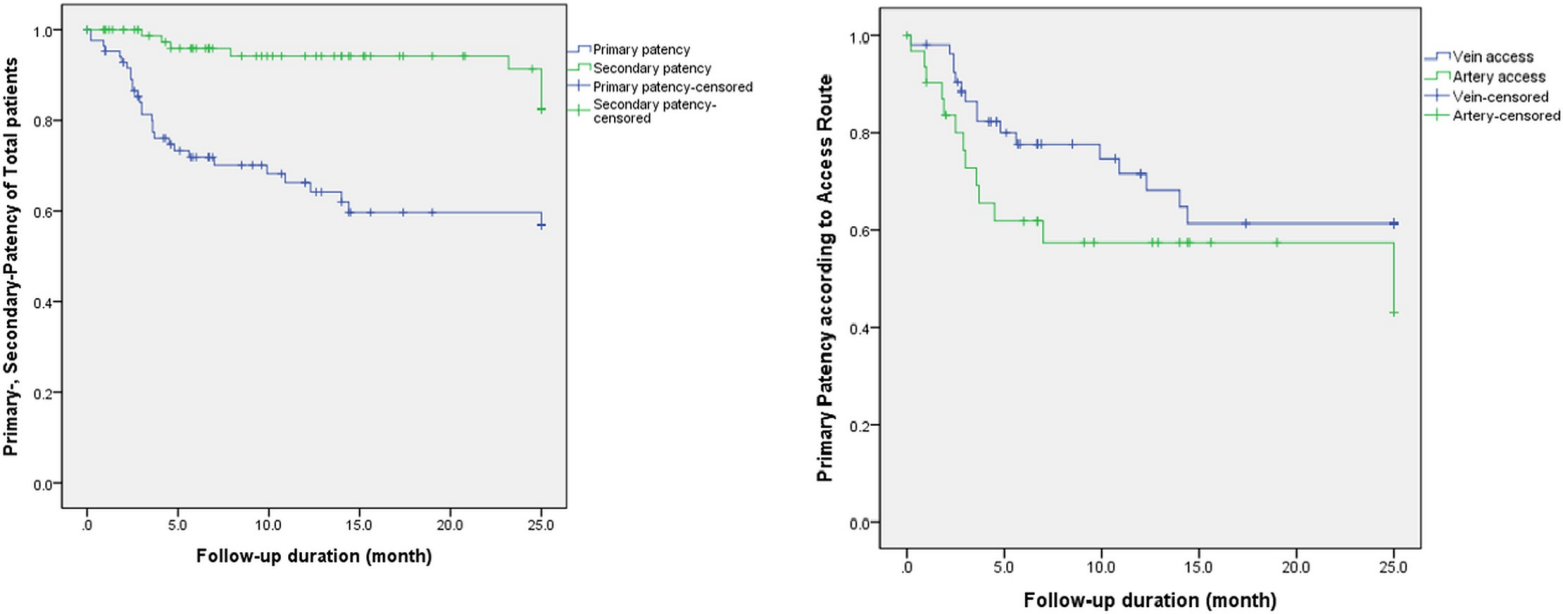
(a) Kaplan-Meier curves showing the primary and secondary patency rates of the non-maturing fistulas treated using percutaneous intervention. (b) Kaplan-Meier curves showing primary patency rates according to the access route after angioplasty. The venous access group shows longer primary patency than the arterial access group, but the difference was not statistically significant.

The procedural details are summarized in Table 2. The anatomical success rate was 98.8% (84/85). One anatomical failure occurred in the arterial access group in a patient with left brachiocephalic AVF. The transradial approach was used because of juxta-anastomotic cephalic vein occlusion. Vascular rupture occurred after dilatation of a 5-mm balloon near the anastomosis. Hemostasis failed even with balloon tamponade, and surgical ligation was performed by a vascular surgeon. The clinical success rate was 83.5% (71/85). The locations of the 123 lesions were as follows: arterial inflow, 19 patients; juxta-anastomosis, 68; peripheral vein, 34; and central vein, 2. Thirty-nine patients had single lesions; and 46, had multiple lesions. The mean lesion length was 5.0±3.8 cm. Seventy-nine patients had stenosed lesions; and 6, occluded lesions. Thrombosis was observed in only 2 patients in the arterial access group. Accessory veins were found in 18.8% (16/85) of the patients, of whom 4 underwent surgical ligation and 5 underwent coil and microcoil embolizations. Seven (78%) of the 9 patients with accessory veins eventually underwent successful dialysis. The mean maximal size of the balloons used for PTA was 5.4±1.1 mm (range, 3–10 mm). Cutting balloons were used in 6 patients. A central venous stent was inserted in 2 patients due to elastic recoil, even after balloon angioplasty. A major complication (technical failure) occurred in 1 patient who underwent surgical ligation. The following minor complications occurred in the remaining 12 patients: hematoma (n = 1), pseudoaneurysm (n = 1), venous dissection (n = 1), and vein rupture (n = 5) in the arterial access group, and hematoma (n = 1) and vein rupture (n = 3) in the venous access group. Eight patients experienced vessel rupture due to balloon dilatation or wire passage and treated successfully using balloon tamponade. Two patients developed regional hematoma at the brachial artery and cephalic vein puncture sites, respectively. They underwent successful hemodialysis after longer compression. The brachial artery was patent on follow-up ultrasonography, and the hematoma spontaneously resolved. The mean procedure time was 31.5±21.1 minutes. The primary and secondary patency rates were 83.9%, 71.9%, and 66.3%, and 98.6%, 95.9%, and 94.2% at 3 months, 6 months, and 1 year, respectively (Fig 3).

**Table 2:** Procedural details.

Characteristic	Vein access (n = 53)	Artery access (n = 32)	Total (n = 85)	P value
Clinical success	45 (84.9%)	26 (81.3%)	71 (83.5%)	0.660
Lesion location	73	50	123	0.188
*Arterial inflow*	15	4	19 (15.4%)	
*Juxta-anastomosis*	40	28	68 (55.3%)	
*Peripheral vein*	17	17	34(27.6%)	
*Central vein*	1	1	2 (1.6%)	
Lesion multiplicity				0.098
*Single*	28	11	39	
*Multiple*	25	21	46	
Lesion length (cm)	4.7±4.0	5.4±3.3	5.0±3.8	0.231
Lesion characteristics				1.000
*Stenosis*	49	30	79	
*Occlusion*	4	2	6	
Accompanying Thrombus	0	2	2	0.139
Accessory veins	9	7	16	0.576
PTA characteristics				
*Maximal balloon size (mm)(range)*	5.3±1.1 (3–10)	5.4±1.2 (4–10)	5.4±1.1 (3–10)	0.996
*Cutting balloon*	1	5	6	0.026*
*Central stent*	1	1	2	1.000
Complication				0.015*
*Major*	0	1(Anatomical failure)	1	
*Minor*	4	8	12	
Procedure time (min)	29.0±18.5	35.7±24.5	31.5±21.1	0.236
Six-month patency				
*Primary patency*	77.6%	61.8%	71.9%	0.150
*Secondary patency*	95.8%	96.0%	95.9%	0.448

Values are presented as number or mean ± SD.

*Statistically significant.

Patients were classified into the venous (53 patients) and arterial access groups (32 patients) according to the approach route. Hypertension was more frequent in the venous access group (P = 0.023). Moreover, the minimal preoperative vein size and increment in the postoperative vein diameter were larger in the venous access group (P = 0.041 and P<0.01, respectively). A cutting balloon was used in 1 patient in the venous access group and in 5 patients in the arterial access group (P = 0.026). The complication rate was higher in the arterial access group (1 major and 8 minor) than in the venous access group (4 minor; P = 0.015). However, no significant difference was observed in the other demographics, fistula characteristics, procedure details, and primary and secondary patency rates between the groups (Tables 1 and 2, Fig 3).

## Discussion

To the best of our knowledge, this is the first study to compare the clinical outcomes and long-term patency of endovascular salvage for non-maturing fistulas according to access route in a large cohort.

The anatomical and clinical success rates were 83.5% and 98.8%, respectively, among 85 patients. One patient experienced vascular rupture near the anastomosis site. Serial and gentle balloon dilatations should be performed, especially in occluded or tight stenotic lesions, as immature veins tend to be vulnerable [15]. Previous studies on PTA for immature fistulas with >30 patients reported clinical success rates of 74%–93% [16–18], and our study showed comparable results. However, the success rates between the venous and arterial access groups were not significantly different.

We achieved primary patency rates of 71.9% and 66.3% at 6 and 12 months, respectively. Secondary interventions such as angioplasty, thrombectomy, and stenting helped to increase the patency rates to 95.9% and 94.2%, respectively. Shin et al. [5], Turmel-Rodrigues et al. [19], and Clark et al. [6] reported 12-month primary patency rates of 61%, 39%, and 34%, respectively, with 12-month secondary patency rates of 82%, 79%, and 75%, respectively. The primary and secondary patency rates in our study were superior to those reported by earlier studies. We believe that the discrepant results are due to the differences in the respective study populations. Turmel-Rodrigues et al. included 17 of 69 immature fistulas with thrombosis and excluded patients with upper arm fistulas, while the present study included only 2 of 85 fistulas with thrombosis and 22 of 85 patients had upper arm fistulas. Dixon et al. [20] reported superior 1-year primary (62% vs. 44%) and secondary patency (69% vs. 52%) for upper arm fistulas as compared with forearm fistulas. Moreover, the three above-mentioned studies did not perform interventions for the accessory veins, which were found in 18.8% (16/85) of the patients and treated surgically or with embolization in our study. Nine of the 16 patients underwent surgical ligation or embolization; thus, hemodialysis was successful in 78% (7/9) of the patients who underwent treatment for the accessory veins. Some researchers have emphasized the importance of treating accessory veins [21, 22]. Beathard et al. [17] used a combination of angioplasty and accessory vein embolization, and achieved a primary patency rate of 68% at 12 months. We believe that an aggressive approach for evaluating non-maturing fistulas with appropriate interventions can improve maturation and patency.

The arterial access group showed significantly smaller minimal preoperative vein size and postoperative increment in diameter than the venous access group. The frequency of cutting balloon use was also higher in the arterial access group. Although not statistically significant, the length and number of lesions were greater in the arterial access group. It seems that the patients who underwent PTA with venous access had more mature fistulas and less severe lesions. We attempted the radial artery approach if the vein was too small to be punctured or the lesions were multiple and severe, which provided favorable outcomes (81.3% clinical success and 61.8% primary patency). Although the retrograde venous approach has traditionally been preferred for mature fistulas, it has some disadvantages. The non-maturing fistula may be flat and difficult to detect without a palpable thrill. Direct catheterization of immature venous walls may induce vessel rupture or focal spasm. Moreover, retrograde venous injection of contrast media may not depict the juxta-anastomotic vein and afferent artery in a considerably occluded lesion. Passing the guidewire and balloon catheter is sometimes difficult because of sharp angulations at the anastomosis site. Recently, the transradial approach was introduced as an alternative for non-maturing fistulas, but only a few studies have focused on this subject [8, 9]. The radial approach has several advantages over the venous approach. First, the radial artery is not compromised in the occluded fistula and is relatively easy to puncture. A single sheath is sufficient to visualize and treat downstream, even multiple or completely occluded, lesions from the radial artery to the central veins. The direction of the lesion has no sharp angulations because the sheath is located distal to the anastomosis site, which facilitates the procedure. Despite these advantages, the relatively small diameter of the radial artery limits the passage of the large introducer sheath for balloon/stent in the central vein or the thrombectomy device for declotting of a large thrombus.

The complication rate was higher in the arterial access group than in the vein access group, which was thought to result from the angioplasty for small, hard lesions that are characteristic of immature fistulas. Puncture site hematoma occurred in 2 patients who underwent brachial artery and cephalic vein punctures. In case of the radial approach, hemostasis was easily and safely achieved with a compression device, without complications such as hematoma, occlusion, or dissection of the radial artery. However, the transradial approach is occasionally not applicable because of the short distance between the puncture and anastomotic sites. The DRA approach through the anatomical snuffbox can be used as an alternative in this case. We performed angioplasty with DRA puncture in 4 patients, all of whom demonstrated anatomical and clinical success. Recently, the distal radial approach has been reported for noncoronary interventions [23, 24]. To the best of our knowledge, management of AVF through the DRA has not been reported yet. The deep radial artery is used in the distal radial approach, which provides enough length for the insertion of the sheath from the anastomosis site and reduces the potential risk of radial artery occlusion and digital ischemia by preserving the blood flow in the superficial palmar arch [25]. Puncture may be difficult owing to the relatively small vascular diameter and weak pulse; thus, all puncture procedures should be performed under ultrasonographic guidance, which requires a longer learning curve than other approaches. We believe that the DRA approach is feasible for non-maturing fistulas, which should be verified with a study with a larger population.

Our study has several limitations. First, its design was retrospective and lacked a control group such as one with patients with mature fistulas. Second, the operator’s preference may have influenced the choice of approach site for PTA. Third, the number of patients who underwent accessory vein treatment or the distal radial approach was relatively small. The clinical values of these interventions remain to be proven with a randomized clinical trial.

In conclusion, an aggressive strategy can salvage most non-maturing fistulas. Transradial and distal radial approaches can be effective and safe alternatives for challenging situations, including very small veins or multiple, diffuse, and occluded lesions.

## Supporting information

S1 File (XLSX)
